# A cross sectional investigation of the development of rhythmic preferences with motor and perceptual tests

**DOI:** 10.1038/s41598-025-87631-2

**Published:** 2025-01-28

**Authors:** Pier-Alexandre Rioux, Simon Grondin

**Affiliations:** Université Laval, Quebec, Canada

**Keywords:** Human behaviour, Statistics

## Abstract

The preferred period hypothesis posits a slowing down of motor and perceptual rhythmic preferences with age, both reflecting an increase in the common internal oscillation period. This study further investigates the preferred period hypothesis by improving the measurement of perceptual rhythmic preferences through two tasks, tempo adjustment and tempo judgment, conducted in auditory and visual modalities. The study was conducted with three groups of children (5–6, 8–9, and 11–12 years old), and a group of young adults (21 to 30 years old) during the same time of the day. Correlational analyses show that the relationship between motor and perceptual rhythmic preferences strengthens between childhood and adulthood. Multilevel analyses reveal a general slowing down of motor and perceptual rhythmic preferences, contingent on the task and modality, with distinct trajectories for males and females. Our study indicate that the range of preferred rhythms narrows with age, becoming less variable in young adulthood, at the expense of a single and consistent, default rhythm. In brief, the preferred period appears only in young adults, with no support for the idea of a slowing down of an internal oscillation that would be reflected by both motor and perceptual rhythmic preferences.

**Supplementary Information:**

The online version contains supplementary material available at 10.1038/s41598-025-87631-2.

## Introduction

The Dynamic Attending Theory proposes a framework for understanding an individual’s engagement with the various rhythmic structures that compose everyday life^1,2^. These environmental rhythms encompass a broad spectrum of both isochronous and nonisochronous temporal configurations. Individuals’ engagement with various external rhythms is understood as an outcome of entrainment, a process characterized by the synchronization of an individual’s internal rhythms, or internal oscillations, with external rhythms^3,4^.

Internal oscillations emanate from an endogenous oscillator, which is conceptualized as a singular periodic biological process^3,4^. Thus, a biological oscillator produces an oscillation period specific to each individual, which is initially independent of external rhythmicity^3,5^. This oscillation period is likely to vary according to individuals’ biological attributes, such as age. In this regard, an increase in the oscillation period with age has been demonstrated in several studies^3,6–10^. The period of the oscillator is commonly measured by the spontaneous motor tempo (SMT)^3,6–10^, which corresponds to the natural, preferential tempo of periodic motor actions^5^. SMT can be measured using a finger-tapping paradigm in which individuals are asked to tap at the rate that seems most natural to them^3,6,9,10^. Inter-taps intervals (ITIs) produced in SMT indicate the period of oscillation.

In that vein, the preferred period hypothesis proposes a general slowdown of preferred event rate across the lifespan^11^. This preferred period hypothesis is characterized by the shared motor and perceptual components underlying the preferred event rate, each slowing down with maturation. The preferred period can be understood as the preferred motor and perceptual event rate, both providing insight into the period of the internal oscillator. The preferred motor tempo is assessed by SMT while the preferred perceptual tempo (PPT) is measured by rating isochronous monotone sequences as “too fast”, “too slow”, or “just right”. First results show that SMT strongly correlates with PPT^11^. The mean periodic duration of the preferred period increases from age 4 (~ 300 ms) to 75 (~ 700 ms)^11^.

The strong relationship between SMT and PPT was confirmed by other researchers^12^. Furthermore, their findings demonstrate that corticomotor excitability is preferentially modulated by the PPT, supporting the notion of an endogenous oscillation shared by auditory-motor coupling. These outcomes align with the substantial multisensory interplay between motor cortex activity and rhythmic perception^13^. In this regard, electroencephalography (EEG) data reveal a correlation between individual’s PPT and the beta frequency of the motor cortex^14^.

Recent studies on young adults have proposed methodological refinements in the measurement of SMT and PPT, leading to a more cautious and conservative interpretation of a preferred period^15,16^. Firstly, when the inter-stimulus interval (ISI) of isochronous sequences presented during PPT measurement are not selected by a participant SMT, no correlation between motor and perceptual rhythmic preferences emerges^15^. According to these researchers, selecting ISI of isochronous sequences in a PPT task based on participants’ SMT might introduce a bias toward the central rhythm used. Moreover, their results show substantial within-subject variability in the measurement of both SMT and PPT^15^. Secondly, an adjustment task with the method of limits approach, instead of a judgment task, was used to measure PPT^16^. Moreover, given that a natural and comfortable rhythm can be subject to interpretation in SMT and PPT measurements, participants were asked to use a counting paradigm to produce and adjust tempi^16^. Results show a strong correlation between counting-SMT and counting-PPT (*r* = .68, *p* < .001^16^. Despite the strength and significance of the correlation, it is not a 1:1 correspondence between motor and perceptual rhythmic preferences. This result suggests that the default motor and perceptual rhythms do not strictly reflect a single common oscillator. Additionally, within-subject variability is much lower in a counting paradigm, but still manifest in the results. These findings also suggest that the idea of a consistent default rhythm should be considered with caution, as intra-individual variability in rhythmic preferences does not point to a single, singular personal tempo. According to these authors^16^, “individuals may have a general tendency toward a particular range of rhythms, yet de-facto behaviour can also be highly flexible, allowing the production of a broad repertoire of rhythms with high accuracy” (p.12).

The general aim of the present study is to deepen, with a cross-sectional approach, the hypothesis of a preferred period in four different age groups, ranging from childhood to adulthood (5–6, 8–9, 11–12, and 21–30 years). Thereby, it is possible to investigate the presence of a crucial point of change in the preferred period during childhood, while comparing with a group of adults. It is recognized that significant changes occur in the rhythmic behavior of children around 8-year-olds^17^. These behavioral changes could be explained by a slowing point in the SMT around 8–9 years of age^11^. Our study allows to further investigate the presence of a change point in terms of inter-group differences with children slightly younger and older than 8–9 years. Furthermore, comparisons with a group of adults will enable us to determine whether the presence of a change point in childhood is indicative of rhythmic preferences similar to those of adults. The present study also introduces a protocol using two distinct tasks to assess PPT. Hence, a judgment task^11,12,15^ and an adjustment task^16^ are employed for the PPT measurement. To the best of our knowledge, this study marks the first instance of employing two types of tasks to assess PPT. In addition, the present study extends the assessment of PPT to the visual modality. On this point, it is well known that timing performances differ between auditory and visual modalities^2,18–21^. From a developmental point of view, the magnitude of the modality effect on temporal acuity is more pronounced in children and diminishes with maturation^19^. Thus, temporal processing in the visual modality entails greater cognitive demand compared to the auditory modality, exerting a more substantial impact on the limited working memory and attentional capacities of children^18,19,21^. However, although the temporal processing varies according to the sensory modality used to mark intervals^18,19,21^, if the hypothesis of a preferred period remains valid, PPT measures should all converge and reflect a consistent internal oscillation, regardless of the task and modality used to assess it. To the best of our knowledge, this study is also the first to include the visual modality in PPT measurements. Furthermore, in order to minimize the effect of fluctuations in participants’ SMT throughout the day^22^, all data were collected during the same period of the day.

The first specific objective of the study is to examine the relationships between the variables that underlie the hypothesis of a preferred period across all age groups. This objective includes investigating the relationships between the different tasks used to measure PPT, in both auditory and visual modalities, as well as their relationships with SMT.

The second specific objective is to determine whether there are differences in motor and perceptual rhythmic preferences, as well as intra-individual variability, between age groups in the various tasks employed. We will examine the influence of trials in SMT, since an individual’s SMT is likely to vary between trials^15,16^. These trial-to-trial variations are considered indicative of intra-individual variability. Additionally, we will investigate the influence of the ISI in standard isochronous sequences in PPT measurements. Variations attributed to the ISI in standard isochronous sequences also serve as an indicator of intra-individual variability in PPT measurement. Furthermore, the influence of sex is also investigated given that, in preschool, rhythmic abilities of females are superior to that of males^23^. These results may indicate different developmental trajectories for males and females, considering that a slower SMT is linked to better rhythmic performances^3,11^.

## Methods

### Participants

The sample (*N* = 70) is composed of three groups of children and one group of young adults: nineteen children aged 5–6 years (5 boys, 14 girls), fifteen children aged 7–8 years (6 boys, 7 girls), sixteen children aged 11–12 years (4 boys, 12 girls) and twenty young adults aged 21–30 years (8 male, 12 female) with a mean age of 25.27 years old (SD = 2.68). An a priori power analysis was performed using G*Power^24^ to estimate the sample size sufficient to achieve a small effect size (0.25)^25^ in a repeated-measure analysis of variance (ANOVA), within-between interaction (1 – β = 0.8; *n*_groups_ = 4, *r =* .50) for a minimum of 3 and a maximum of 90 measurements. The power analysis revealed that a total sample size of 12 to 60 participants was sufficient to achieve a small effect size. Children were recruited from their respective elementary school in Quebec City, with prior agreements established with the school services, principals, and teachers. Children were recruited through informational flyers distributed to their parents by their respective teachers. Parents who agreed to their child’s participation formally signed the informed consent form. Additionally, children provided their assent before starting the experiment. Adults were recruited through electronic advertisement and were students at Laval University. Children and adults had no psychological or neurological disorders. None of the participants had musical concentration at school and/or played an instrument for more than five hours per week. Children received school supplies as compensation (pencil, eraser, highlighter, notebook), while adults received $15 CAD. This study was approved by an independent local ethics committee (Comité d’éthique de la recherche de l’Université Laval: #2022 − 480 / 21-02-2023). The research was conducted in accordance with the relevant guidelines and regulations (e.g., Declaration of Helsinki).

### Measures and procedure

The data presented in this article are part of a larger-scale data collection project. For children, the sociodemographic questionnaire was completed by a parent and returned with the signed consent form. For adults, the sociodemographic questionnaire was administered following the informed consent form. The participants completed the tasks in the following order SMT (Trial 1), judgment and adjustment tasks in auditory modality, SMT (Trial 2), judgment and adjustment tasks in visual modality and SMT (Trial 3). A practice trial was conducted each time a task was performed for the first time.

Participants carried out the experiment between 1:00 pm and 3:30 pm in a quiet room. The experiment was conducted on a Lenovo ThinkPad P15s with a 15.6” screen, a resolution of 3840 × 2160 pixels and a refresh rate of 60 Hz. Auditory tempi were delivered through two speakers placed on either side of the computer and facing the participants. The experiment was set up and run using E-Prime 3.0 software^26^. Responses were collected using the Chronos^®^ module. In the case of children experiment, a turtle sticker was placed on the key at the left end of the module, while a rabbit sticker was placed on the key at the right end. In the case of adults’ experiment, a sticker with a “-” sign was placed on the key at the left end of the module, while a “+” sign was placed on the key at the right end of the module.

#### SMT

This measure was captured by digital tapping at a pace that felt natural and comfortable for participants. In each trial, participants were asked to produce 31 ITIs. The practice trial consisted in the production of 10 ITIs.

For each trial performed by a participant, the first ITI was systematically removed. Thus, 30 intervals were included in the analyses. In regard to outliers, within each trial, ITIs that were three times greater or less than the median value were removed using the *outliers_mad* function in the *Routliers* package for R^27^. One participant in the 5–6 age group declined to perform the first of three trials. Therefore, 222 out of 6300 ITIs were removed from the analyses, representing 3.52%. The mean ITI and coefficient of variation (CV: standard deviation divided by mean) for each and all trials were calculated and retained for the analyses.

#### Adjustment task—auditory and visual modalities

**Fig. 1:**
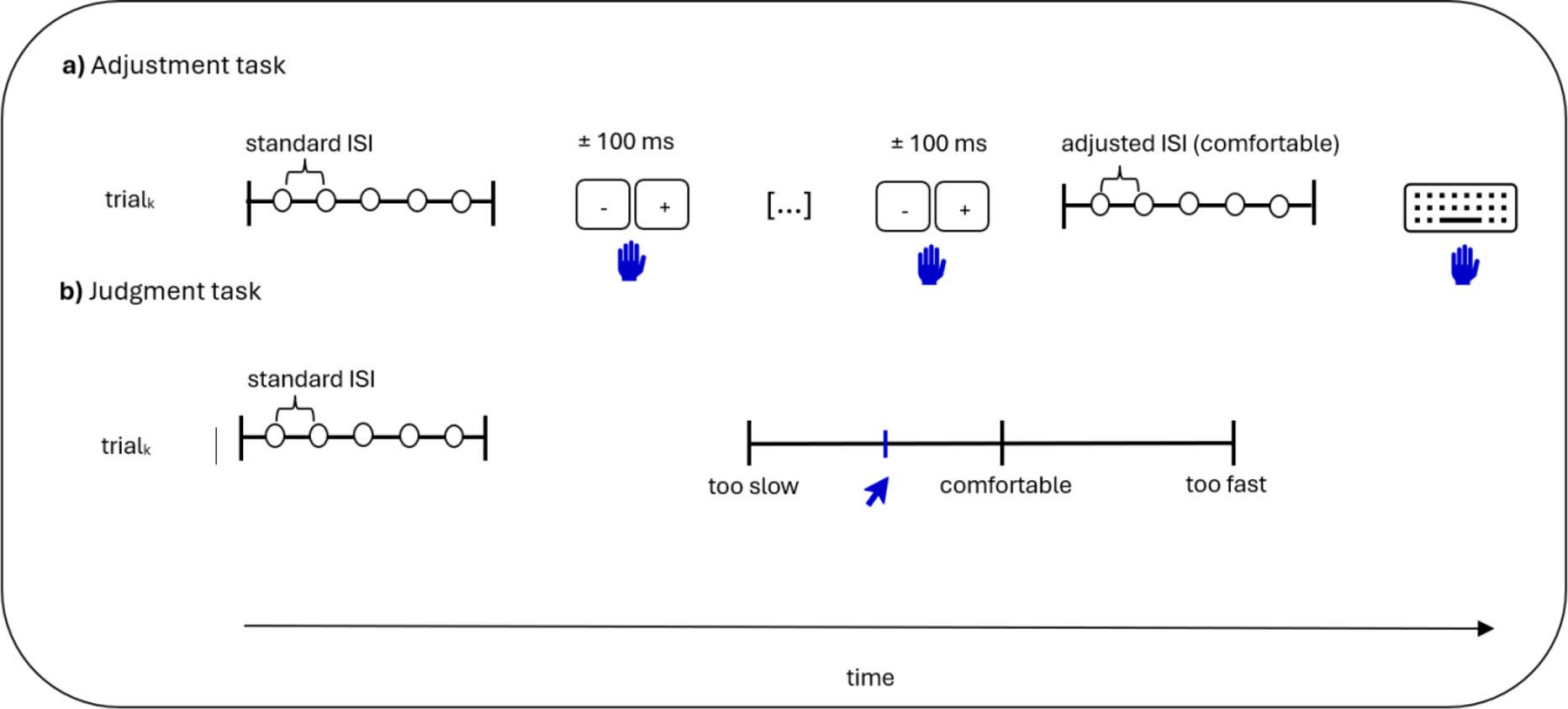
Experimental design of preferred perceptual tempo (PPT) measurements in (**a**) Adjustment task and (**b**) Judgment task. Blue icons (hands and pointer) represent participants’ interventions to indicate their PPT. ISI, inter-stimulus interval.

Participants adjusted standard ISI of isochronous sequence (speed up or slow down with 100-ms steps) to a tempo that felt natural and comfortable to them. Specifically, participants accelerated or decelerated a default isochronous sequence (standard ISI) until the adjusted ISI of the isochronous sequence seemed natural and comfortable. The adjusted isochronous sequence was presented after each 100-ms adjustment. Participants were free to accelerate or decelerate the tempo as they liked. Tempo acceleration was achieved using the button on the right of the Chronos module, while tempo deceleration was achieved using the button on the right of the Chronos module. Once the tempo felt natural and comfortable, participants pressed the space bar on the computer keyboard. No time limit was given for the task. The experimental paradigm for this task is illustrated in Fig. 1a. For the auditory modality, participants adjusted isochronous sequences composed of five intervals delimited by 440-Hz tones delivered during 500 ms at 60 dB. For the visual modality, participants adjusted isochronous sequences composed of five intervals delimited by a 500-ms presentation of a black circle on a gray background. For both modalities, standard ISIs were 200 ms, 300 ms, 400 ms, 500 ms, 600 ms, 700 ms, 800 ms, 900 ms, 1000 ms and 1200 ms. Standard ISIs were selected based on a previous study^16^, although we retained a larger number of standard ISIs and slightly restricted the range for faster tempi because of the faster rhythmic preferences in childhood^3,11^. The presentation order of isochronous sequences was randomized. Each standard ISI of isochronous sequence was adjusted once in each modality. The practice trial in both modalities required adjusting an isochronous sequence of 600-ms ISI (which corresponds to the median of standard ISIs).

For each auditory and visual modality tests performed by a participant, adjustments that were three times greater or less than the median value were removed using the *outliers_mad* function in the *Routliers* package for R^27^. Therefore, 26 out of 700 adjustments (3.71%) in the auditory modality were removed from the analyses, while 33 out of 700 adjustments (4.71%) in the visual modality were also removed from the analyses. The mean adjusted ISI and the CV were calculated and retained for subsequent analyses.

#### Judgment task—auditory and visual modalities

PPT was measured by rating isochronous sequences on a VAS ranging from “too fast” (-10) through “comfortable” (0) to “too slow” (10). The experimental paradigm for this task is illustrated in Fig. 1b. For children, an image of a rabbit was at the top of value 10 (i.e., at the right end of the VAS), while an image of a turtle was at the top of value − 10. For adults, “too fast” was indicated at the right end of the VAS, “too slow” was indicated at the left end, and “comfortable” was indicated in the center. For auditory modality, participants judged isochronous sequences composed of five intervals delimited by 440-Hz tones delivered during 500 ms at 60 dB. For visual modality, participants judged isochronous sequences composed of five intervals delimited by the brief presentation of a black circle on a gray background (i.e., of 500 ms). For both modalities, standard ISIs were 200 ms, 300 ms, 400 ms, 500 ms, 600 ms, 700 ms, 800 ms, 900 ms, 1000 ms and 1200 ms. Standards ISIs are the same as those for the adjustment task. Isochronous sequences were presented in random order. Each standard ISI was judged once in each modality. The practice trial in both modalities required judging a 600-ms ISI isochronous sequence (which corresponds to the median of standard ISIs). No data was considered as an outlier. The intercept was calculated and retained for each participant in both modalities.

### Statistical analyses

All analyses were performed using IBM SPSS Statistics version 29 software^28^ and R Project version 4.3.1^29^ (ɑ = 0.05).

First, Pearson correlations were conducted to evaluate the magnitude and statistical significance of the associations among the variables within each and all age groups. Correlational analyses were performed on the mean ITI obtained in SMT, the mean adjusted ISI in auditory and visual adjustment task, as well as on the mean intercept derived from auditory and visual judgment task.

Secondly, multilevel mixed models (MLMs) were conducted to capture the effect of age group, sex, trial (in the SMT task), and standard ISI of isochronous sequences (in the adjustment and judgment tasks). MLMs were chosen due to the hierarchical structure of participants’ repeated measures across SMT, adjustment and judgment tasks. In this regard, MLMs are a more powerful and flexible alternative to repeated-measures ANOVAs^30,31^. Multilevel modeling is useful, notably because it includes random intercepts due to the increased Type I error rate that occurs in hierarchical data when groups exhibit differences in the average value of the dependent variable^32^. Additionally, MLM allows variation of slopes (relationships between independent and dependent variables) across different levels of a model’s structure. Moreover, multilevel modeling enables the inclusion of predictors on each of the levels specific to a model and is expressed as a set of regression equations. In our study, a two-level structure model was conceived to identify the effect of age group, sex, trial (SMT task) and standard ISI of isochronous sequences (adjustment and judgment tasks) on variables underlying the preferred period hypothesis. As of level-two predictors, participant was included as a random effect, while the group and the sex of the participant was included as a fixed effect. For level-one predictor, the three trials in the SMT task and the ten standard ISIs of isochronous sequences in the adjustment and judgment tasks were included as fixed effects. The standard ISIs were centered at 200 ms for theoretical reasons (an ISI at 0 ms is theoretically useless) and, by the same rationale, to simplify interpretation of the results. The intercepts-only model (Baseline) only included participant as a random effect and was conducted on each preferred period measures. Chi-square analyses were carried out to compare the deviance fit index between the two models for each measure (Model vs. Baseline).

Regarding variables derived from SMT, multilevel analyses were conducted on ITIs and CVs. For the adjustment task, multilevel analyses were conducted on the adjusted ISIs. As for the judgment task, the MLMs was run on each value indicated on the VAS for each isochronous sequence.

Additionally, an analysis of variance (ANOVA) was conducted on CVs obtained in the adjustment tasks to investigate whether there were differences in variability between age groups. Subsequently, post-hoc analyses were carried out using the Bonferroni multiple comparison method.

No analysis was performed on intra-individual variability in the judgment task, as each judgment was made only once for each standard ISI.

## Results

**Fig. 2:**
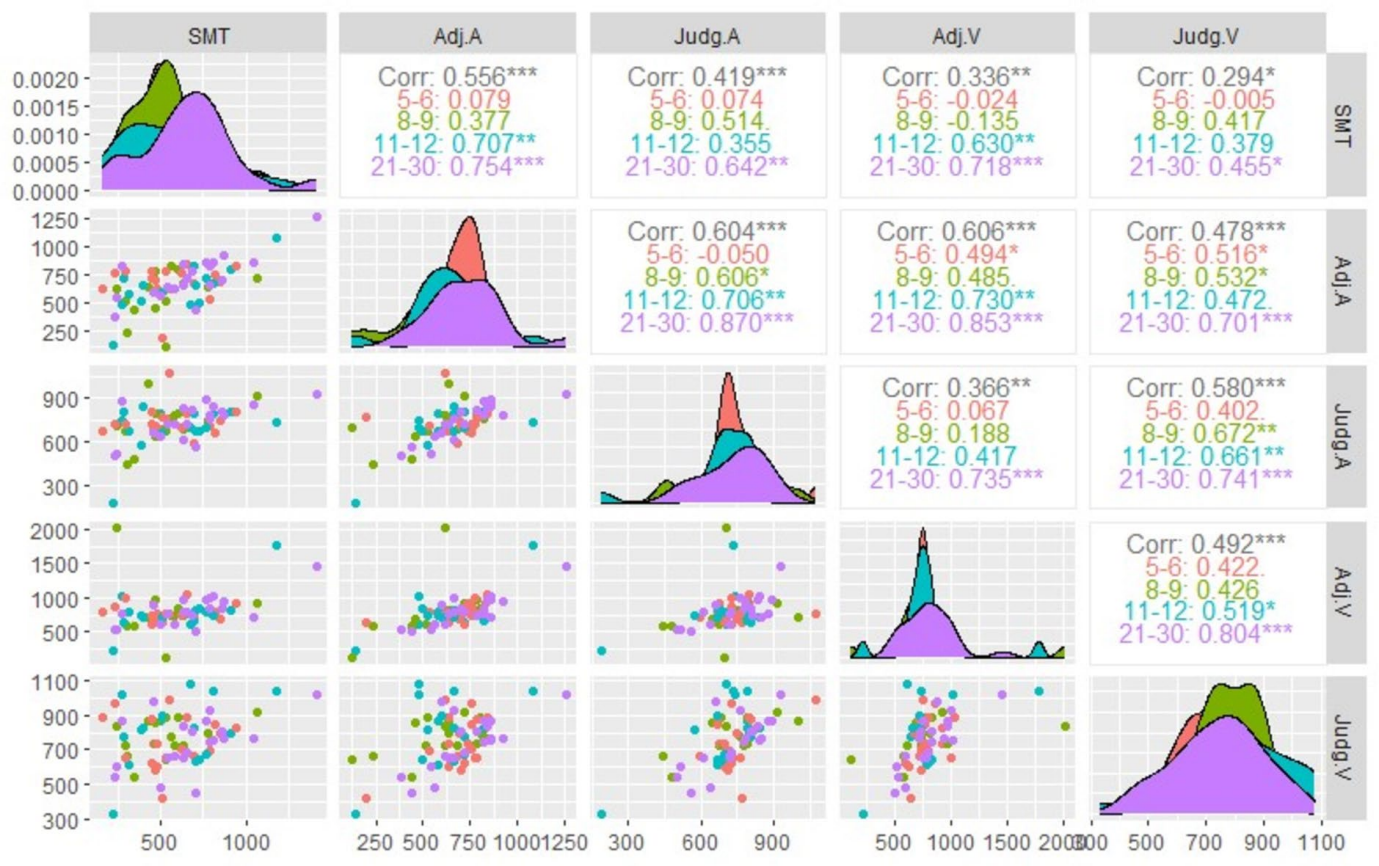
Density curves, scatter plots, and correlation matrix of mean ITI (SMT task), adjusted ISI (adjustment task), and intercept (judgment task) for each and all age groups. Corr. indicates the correlation coefficient for all age groups. SMT, Spontaneous Motor Tempo; Adj.A, Adjustement Auditory; Adj.V, Adjustment Visual; Judg.A, Judgment Auditory; Judg.V, Judgment Visual. *p < .05. **p < .01. ***p < .001.

Table 1 displays the mean ITI and CV obtained in SMT task, the mean adjustment and CV obtained in adjustment task and the mean intercept derived from the judgment task, categorized by sex within each and all age groups. Figure 2 shows the density curves for mean ITI in the SMT task, mean adjusted ISI in adjustment tasks, and mean intercept in judgment tasks for each age group.

**Table 1:** Mean inter-tap interval (ITI) and coefficient of variation (CV) obtained in the SMT task, mean adjusted inter-stimulus interval (ISI) and CV obtained in adjustment tasks, and mean intercept obtained in judgment tasks categorized by sex within each and all age groups.

Age group (years)	*n*	SMT		Adj.A.		Adj.V.		Judg.A.	Judg.V.
		M	CV	M	CV	M	CV	M	M
5–6	19	550	37	688	39	784	36	740	744
M	5	466	38	649	39	741	33	746	656
F	14	580	37	702	40	799	37	737	776
8–9	15	524	28	574	28	790	26	719	772
M	6	461	35	546	30	644	29	691	740
F	7	566	23	593	26	888	24	737	794
11–12	16	584	20	626	24	791	24	696	804
M	4	544	22	596	27	620	21	623	643
F	12	597	20	636	22	849	24	720	858
21–30	20	674	8	727	16	813	15	740	743
M	8	826	5	811	12	862	12	765	746
F	12	550	10	659	20	773	17	721	739
Total	70	588	23	661	27	795	25	725	764
M	23	612	22	675	25	742	22	719	709
F	47	575	23	653	28	823	26	729	792

SMT, spontaneous motor tempo; Adj.A., Adjustment Auditory; Adj.V., Adjustment Visual; Judg.A., Judgment Auditory; Judg.V., Judgment Visual.

### Interrelationships between variables underlying the preferred period

Figure 2 provides a correlation matrix between the preferred period measurements. Correlation coefficients are provided for each and all age groups. The correlations carried out across all age groups demonstrate that the relationships between all the variables are significant. Furthermore, the matrix reveals that relationships between the variables underlying the preferred period become stronger and exhibit greater statistical significance for the 21–30-year-olds than for the 5–6-year-olds. This result is especially evident in terms of associations between all PPT variables and SMT. Additionally, relationships between adjustment and judgment, within the same modality, get stronger and more significantly related for the 21–30-year-olds than for the 5–6-year-olds. Concurrently, correlations between the two modalities of the same task (adjustment or judgment) increase and get more significant from 5–6-year-olds to 21-30-year-olds. Except for the relationship between SMT and visual judgments, correlations between the variables are all strong and significant in young adults. In contrast, among 5-6-years-old, only one relationship is significant, namely between auditory adjustment and visual judgment. Meanwhile, the number of significant relationships increases in the 8–9 and 11–12 age groups. However, the strength of the relationship between the variables does not indicate that relationships get stronger for the 11–12 years old than the 8–9 years old. Meanwhile, correlations between measurements across all groups are consistently stronger for men than for women (see Figure S1).

### Multilevel modeling: implications of age group, sex, and trial/ISI in variables underlying the preferred period

#### Spontaneous motor tempo

Results of MLMs performed on ITIs and CVs obtained in SMT are presented in Table 2. Both chi-square analyses show a better fit for the two-level structure model (Model) used for each variable, although there remains a significant residual variance due to participants that is unexplained.

**Table 2:** Model parameters (β) and goodness of fit for age group, trial, and sex in SMT. Standard errors are in parentheses.

Effect	ITI		CV	
	Baseline	Model	Baseline	Model
Fixed effects				
Intercept	587.70*** (29.44)	846.26*** (79.325)	22.87***(1.96)	10.16** (4.05)
Age group				
5–6		− 408.08*** (132.50)		28.34*** (5.38)
8–9		− 397.34*** (125.25)		13.85* (5.96)
11–12		− 244.55^†^ (142.69)		10.82^†^ (5.59)
21–30		0		0
Trial				
1		− 30.53* (13.08)		1.51 (2.84)
2		− 30.84* (13.05)		− 0.98 (2.84)
3		0		0
Sex				
F		− 298.86** (106.73)		− 6.51 (5.77)
M		0		0
Random effects				
Residual variance				
Participant	60231.35*** (10181.71)	55715.69*** (10081.40)	245.58*** (45.29)	129.96*** (27.41)
Model statistics				
Goodness of fit				
* Deviance*	81,142	80,956	1649	1579
χ^2^ (df)		186.09 (17)		7051.00 (17)
* p*		< 0.001		< 0.001

ITI, inter-tap interval; CV, coefficient of variation. ^†^*p* ≤ .10. *p < .05. **p < .01. ***p < .001..

**Fig. 3:**
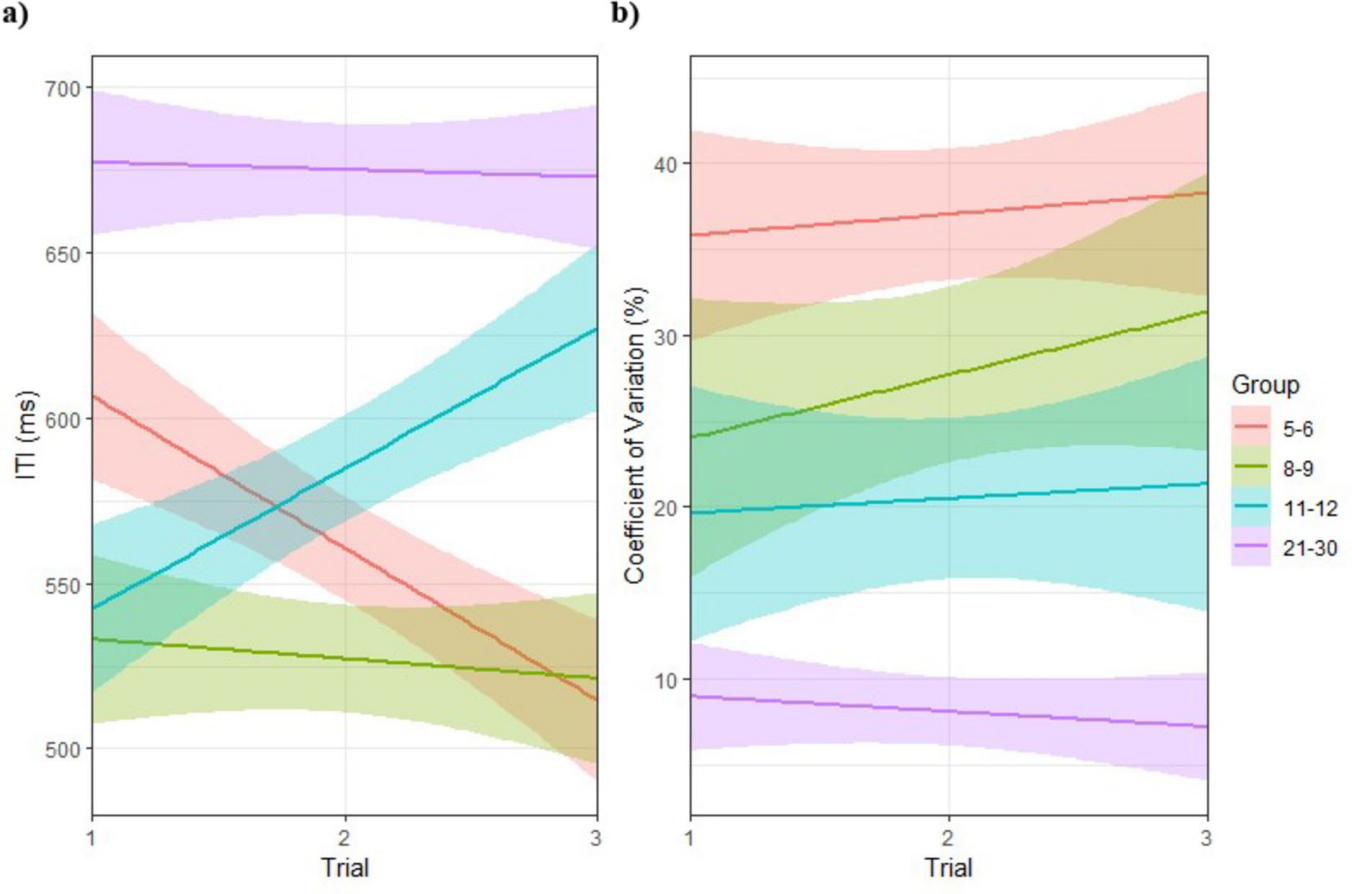
(**a**) ITIs as a function of the trial and (**b**) CVs as a function of the trial in SMT for each age group. Participants aged 5–6, 8–9, 11–12 and 21–30 are represented by the color red, green, blue, and purple, respectively.

Regarding ITI, results indicate that the 5–6- (*p* = .003) and 8–9-year-olds (*p* = .002) have a significantly faster SMT than the 21–30-year-olds. Moreover, for all age groups, the ITIs produced in the first two trials are significantly shorter than in the third trial (*p* < .001). However, the significant interaction between age group and trial (*p* < .001) indicates that the 5–6-year-olds are significantly faster in the first two trials (*p* < .001). Regarding sex differences, females produced shorter ITIs. However, a significant interaction between group and sex (*p* = .05) reveals that females are significantly slower than males at ages 5–6 (*p* = .019) and 8–9 (*p* = .024). Furthermore, the significant interaction between sex and trial (*p* < .001) shows that females are significantly faster in the first trial than in the third (*p* < .001). Figure 3a illustrates the regression line of ITIs as a function of trial for each age group.

Regarding CV results, 5–6-year-olds (*p* < .001) and 8–9-year-olds (*p* = .023) exhibit significantly greater within-subject variability than 21–30-year-olds, while 11–12-year-olds tends towards greater within-subject variability than 21–30-year-olds (*p* = .056). Additional analyses carried out by changing the reference group in the three-level structure model also show that children in the 5-6 age group are significantly more variable than the children in the 8-9 (*p* = .001) and 11-12 (*p* = .013) age groups. Figure 3b shows the regression line of CVs as a function of trial for each group.

#### Preferred perceptual tempo

Results of MLMs conducted on adjustment and judgment tasks are presented in Table 3. All chi-square analyses show a better fit for the two-level structure model used for each variable, although there also remains an unexplained significant residual variance due to participants.

**Table 3:** Model parameters (β) and goodness of fit for age group, trial, and sex in adjustment and judgment tasks in both auditory and visual modalities.

Effect	Adj.A.		Adj.V.		Judg.A.		Judg.V.	
	Baseline	Model	Baseline	Model	Baseline	Model	Baseline	Model
Fixed effects								
Intercept	660.68*** (22.65)	511.33*** (63.18)	795.94*** (31.65)	631.88*** (88.50)	1.13*** (0.21)	9.43*** (0.89)	1.88*** (0.26)	10.24*** (0.96)
Group								
5–6		-379.9*** (84.52)		-447.08*** (118.15)		4.28*** (1.17)		0.39 (1.25)
8–9		-214.53* (92.37)		-107.30 (129.78)		3.70** (1.26)		-1.05 (1.35)
11–12		-124.06 (87.00)		-127.49 (121.35)		1.64 (1.22)		0.18 (1.30)
21–30		0		0		0		0
ISI		0.17*** (0.036)		0.16*** (0.41)		− 0.01*** (0.001)		− 0.01*** 0.001
Sex								
F		261.70** (88.23)		213.51^†^ (125.80)		0.99 (1.08)		− 0.71 (1.19)
M		0		0		0		0
Random effects								
Residual variance								
Participant	32591.21*** (6028.67)	31718.65*** (6033.65)	65724.33*** (11766.94)	69181.61*** (12862.92)	0.58 (0.57)	2.18*** (0.57)	2.99*** (0.83)	3.23*** (0.77)
Model statistics								
Goodness of fit								
* Deviance*	9062	8706	9168	8822	4289	3666	4094	3709
χ^2^ (df)		355.96 (12)		345.12 (12)		623.56 (12)		385.39 (12)
* p*		< 0.001		< 0.001		< 0.001		< 0.001

Standard errors are in parentheses. Adj.A., Adjustement Auditory; Adj.V., Adjustment Visual; Judg.A., Judgment Auditory; Judg.V., Judgment Visual; ISI, inter-stimulus interval. ^†^*p* ≤ .10. *p < .05. **p < .01. ***p < .001..

#### Auditory adjustment

**Fig. 4:**
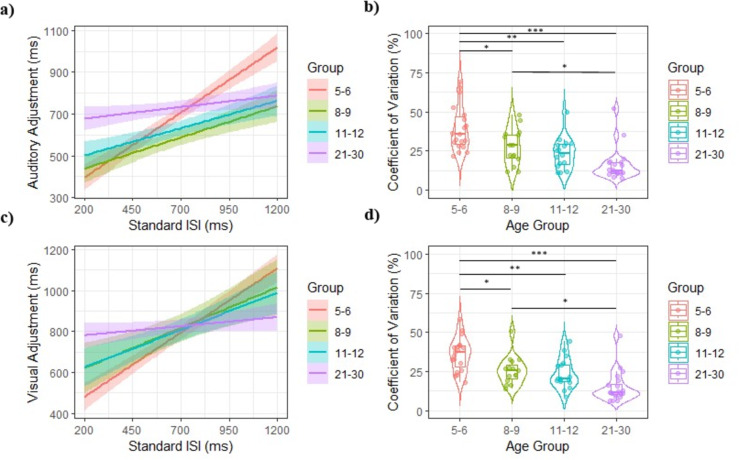
(**a**) Auditory adjustments as a function of standard ISI, (**b**) violin plot including boxplot of participants’ coefficients of variation in the auditory adjustment task for each age group, (**c**) visual adjustments as a function of standard ISI and (**d**) violin plot including boxplot of participants’ coefficients of variation in the visual adjustment task for each age group. Participants aged 5–6, 8–9, 11–12 and 21–30 are represented by the color red, green, blue, and purple, respectively. *p < .05. **p < .01. ***p < .001.

Results show that 5–6-year-olds (*p* < .001) and 8–9-year-olds (*p* = .022) have significantly shorter adjusted ISI than 21–30-year-olds (*p* = .022). Changing the reference group in Model shows that 5–6-year-olds also has significantly shorter adjusted ISI than 11–12-year-olds (*p* = .003). In addition, a sex effect shows that females have significantly shorter adjusted ISI than males (*p* = .004). However, Table 1 indicate that males have shorter adjusted ISI than females in all age groups, except for young adults, for whom the trend is reversed. In parallel, there is an effect of standard ISI (*p* < .001), indicating that the shorter the standard ISI, the shorter the adjusted ISI. This effect is significantly more pronounced for 5–6-year-olds (*p* < .001), 8–9-year-olds (*p* < .001), and 11–12-year-olds (*p* = .016) than for 21–30-year-olds. Figure 4a illustrates the regression line of adjusted ISI as a function of standard ISI for each age group. The influence of standard ISI on adjusted ISI is particularly important in the 5–6 age group.

In regard to CV, ANOVA shows a significant effect of the age group *F*(3, 66) = 13.30, *p* < .001, ƞ^2^ = 0.376. Post-hoc analyses show that 5–6-year-olds are significantly more variable than 8–9-year-olds (*p* = .03), 11–12-year-olds (*p* = .001), and 21–30-year-olds (*p* < .001). Moreover, 8–9-year-olds are also more variable than 21–30-year-olds (*p* = .030). Figure 4b illustrates the greater within-subject variability in the 5–6 age groups.

#### Visual adjustment

Results show that 5–6-year-olds have shorter adjusted ISI than 21–30-year-olds (*p* < .001). Moreover, there is a significant effect of standard ISI (*p* < .001), so that the shorter the standard ISI, the shorter the adjusted ISI. This effect is significantly more pronounced for 5–6-year-olds, 8–9-year-olds and 11–12-year-olds than for 21–30-year-olds (*p* < .001). Figure 4c illustrates the regression line of adjusted ISI as a function of standard ISI for each age group.

In regard to CV, ANOVA shows a significant effect of the age group *F*(3, 66) = 14.80, *p* < .001, ƞ^2^ = 0.402. Post-hoc analyses show that 5–6-year-olds are significantly more variable than 8–9-year-olds (*p* = .024), 11–12-year-olds (*p* = .003) and 21–30-year-olds (*p* < .001). Moreover, 8–9-year-olds are also more variable than 21–30-year-olds (*p* = .013). Figure 4d illustrates the greater within-subject variability in the 5–6 age group.

#### Auditory judgment

**Fig. 5:**
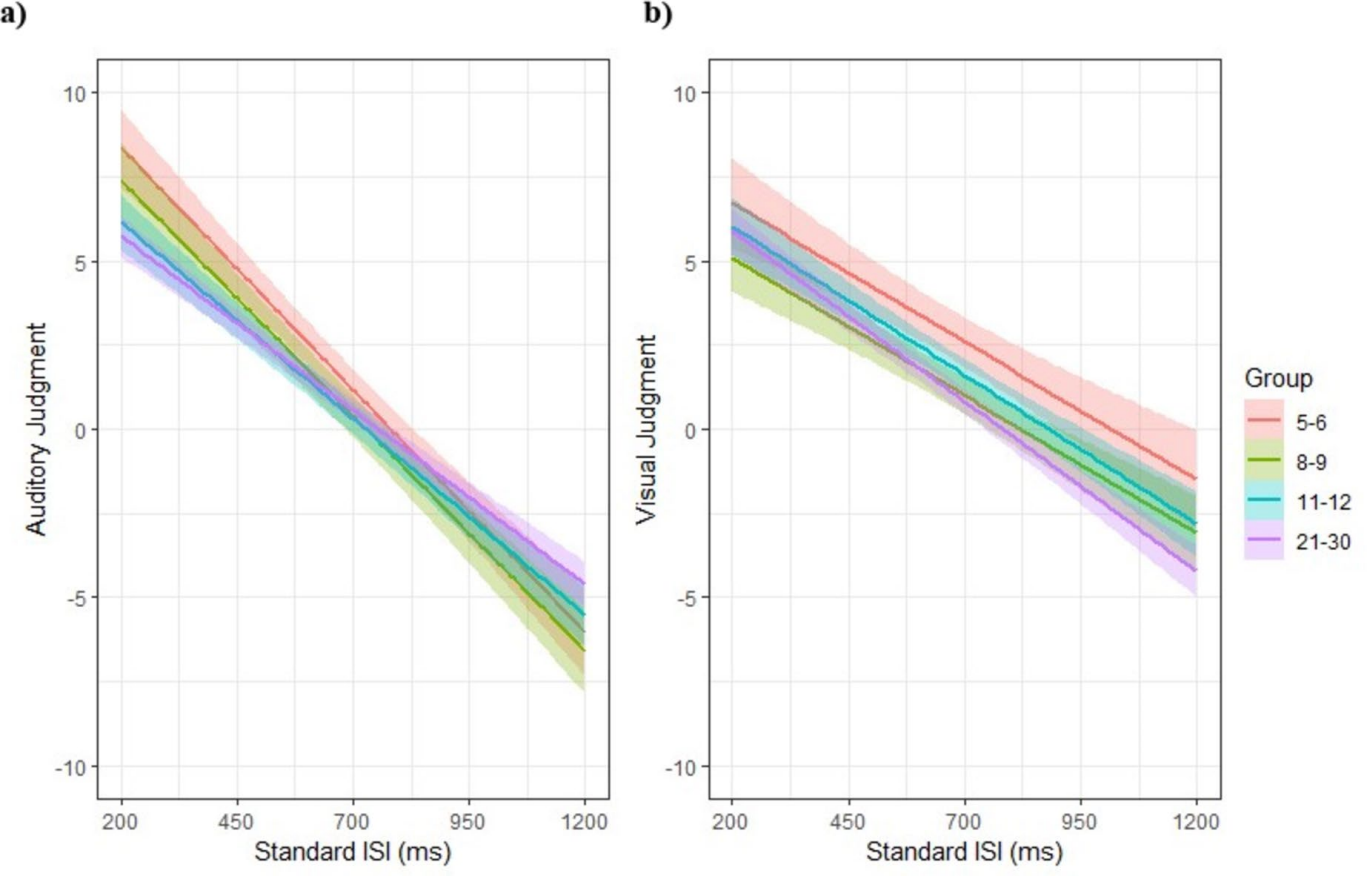
(**a**) Auditory judgment and (**b**) visual judgment as a function of standard ISI. Participants aged 5–6, 8–9, 11–12 and 21–30 are represented by the color red, green, blue and purple, respectively.

Results demonstrate that 5–6-year-olds (*p* < .001), and 8–9-year-olds (*p* = .003) have a significantly faster natural and comfortable tempo than 21–30-year-olds. Changing the reference group in Model also shows that 5–6-year-olds have a significantly faster natural and comfortable tempo than 11–12-year-olds (*p* < .001). Moreover, the results show a significant effect of standard ISI (*p* < .001), indicating that the longer the standard ISI, the slower tempo was perceived. This effect is significantly more pronounced for 5–6-year-olds, and 8–9-year-olds than for 21–30-year-olds (*p* < .001). Figure 5a illustrates the regression line of auditory judgment as a function of standard ISI for each age group.

#### Visual judgment

Results indicate no significant difference between age groups. However, there is a significant effect of standard ISI (*p* < .001). The increased standard ISI led to a perception of a slower tempo. Figure 5b illustrates the regression line of visual judgment as a function of standard ISI for each age group.

## Discussion

The aim of the study was to further investigate the preferred period hypothesis, which posits that motor and perceptual rhythmic preferences, both reflecting a common endogenous oscillation, slow down with age, with a marked slowdown around 8–9 years of age. To this end, while retaining the SMT as a measure of motor rhythmic preferences, we employed two different tasks to assess the PPT, in addition to extending the latter to the visual modality in four age groups ranging from childhood to adulthood. Specifically, adjustment and judgment tasks involving isochronous sequences were employed in the measurement of PPT in the auditory and visual modalities in three groups of children aged 5 to 6, 8 to 9, and 11 to 12, and in adults aged 21 to 30. All participants carried out the experiment during the same time of day.

As a first step, correlations were performed to investigate the relationships between motor and perceptual rhythmic preferences, depending on the PPT measure and modality. The statistical significance of the correlations performed on the whole sample supports the hypothesis of a preferred period. Our results confirm the presence of a preferred event rate common to both motor and perceptual components^11,12^. In contrast with recent study^15^, the correlations between motor and perceptual components are significant even if the isochronous sequences presented in the PPT measurement are not selected based on a participant’s SMT. Nevertheless, our study shows that these relationships vary by age group. In this regard, among 5-6- and 8-9year-olds, no association is observed between motor and perceptual rhythmic preferences, whereas relationships become strong and statistically significant among 21- to 30-year-olds. These findings refine the hypothesis of a preferred period, suggesting that the associations between motor and perceptual preferences only appears in young adulthood.

The correlations carried out on the entire sample demonstrate that the association between motor and perceptual components remains consistent regardless of the task or modality used to measure the PPT. However, correlational analyses performed on each age group show that motor and perceptual rhythmic preferences are significant only in the 21–30 group when the PPT is measured using a judgment task. In this latter case, the relationship between motor and perceptual rhythmic preferences emerges earlier in childhood when the PPT is measured using the adjustment task (11 to 12), in both the auditory and visual modalities. Meanwhile, the results indicate that the statistical significance of the association between the tasks used to measure the PPT within the same modality also appears with age. In this regard, the relationship between PPT measures appears earlier in the auditory modality (8–9 years) than in the visual modality (11–12 years). These findings highlight that the task and modality employed to measure perceptual rhythmic preferences have an impact on the age at which they significantly correlate with motor rhythmic preferences. Specifically, associations with rhythmic motor preferences appear later when both the visual modality and the judgment task are employed to measure PPT.

Secondly, we used multilevel modeling to investigate the slowing of motor and perceptual rhythmic preferences, as suggested by the preferred period hypothesis^11^. Regarding SMT, our study confirms the slowing down of motor rhythmic preferences with aging^3,6–10^. Our results indicate that children aged 5–6 and 8–9 exhibit a faster motor rhythm preference than young adults, which aligns with the proposed slowing down of the preferred rate event^11^. However, our study highlights the significant influence of sex in the context of SMT slowing down. Specifically, females demonstrate slower performance than males at ages 5–6 and 8–9, but this trend reverses in young adulthood, with females exhibiting faster performance. Table 1 provides a representation of the average slowing down of SMT in males, while the data for females do not show a pattern of slowing down. On the one hand, our study converges with the differences observed between 5–6-year-old males and females in rhythmic motor skills^23^. On the other hand, it more broadly points to sex differences in rhythmic motor preferences, as well as different developmental paths between males and females. However, it is noteworthy that there is greater inter-individual variability in the young adult group. It is possible that the small sample of female young adults, combined with this greater inter-individual variability observed in this age group, does not allow the expected slower period effect to be reproduced. In this regard, the mean SMT period observed in female young adults is faster than the averages observed in adults in previous studies^3,11^. Nevertheless, our study suggests that the effect of sex should be considered with caution when interpreting changes in oscillator period with age, since a general effect is observed across all age groups, and the trajectories reported in this study are distinct for males and females. The differences observed in the correlations between motor and perceptual rhythmic preferences in males and females also contribute to consider the effect of sex in further studies on rhythmic preferences.

Regarding PPT, the results confirm a general slowing down of perceptual rhythmic preferences with aging^11^, except for one measure. Although children aged 5 to 6 years predominantly have a faster PPT than young adults, the task and modality used to measure the PPT is important, as this slowdown is not confirmed in the visual judgment task. Furthermore, in the auditory modality, children aged 8 to 9 also exhibit a preference for a faster PPT than young adults, which contrasts with the results in the visual modality. Taken together, these intergroup differences suggest different developmental trajectories in the preferred event rate, these trajectories relying on the choice of task and sensory modality. In parallel, the sex factor also leads to consider with caution the slowing down of the PPT in the auditory adjustment task. The results show that males exhibit a faster PPT than females during childhood, but this finding is reversed in adulthood, as is the case for the SMT. In fact, Table 1 also shows no evidence of a slowing down of the PPT for females in the auditory adjustment task. As in SMT results, it is also possible that the small sample of female young adults and the greater inter-individual variability leads to cautious consideration of the divergence from the expected slowdown in rhythmic preferences among females.

In line with recent studies^15,16^, our results indicate significant intra-individual variabilities across all age groups. In the case of SMT, participants’ exhibit different preferred motor tempo in various trials. Similarly, judgments and adjustments are influenced by the standard ISI in isochronous sequences involved in PPT measurements. Specifically, motor rhythmic preferences significantly vary over a short period of time (between trials), while the presentation of a tempo significantly affects the indicated perceptual preference. This effect is stronger in 5-6- and 8–9-year-olds than in 21–30-year-olds. Consequently, the present findings do not point toward the existence of a single and consistent default rhythm; they rather suggest a general tendency towards a certain range of preferred motor and perceptual rhythms^16^. This range seems even greater and more flexible in young children, as their motor rhythmic preferences are more variable from trial to trial, and their perceptual rhythmic preferences are more influenced by the prior presentation of a tempo. Meanwhile, for both SMT and PPT measures, children aged 5 to 6 and 8 to 9 exhibit greater intra-individual variability, within the same task, compared to young adults. This intra-individual variability is a consistent and robust result across the various measurements of the preferred period. In this regard, these results also highlight that the range of preferred rhythms is particularly wide and flexible in young children.

Altogether, the present study reveals that the relationship between motor and perceptual preferences emerges with age, being absent in young children and strong in young adults. Our results indicate that there is no common endogenous oscillation reflected by motor and perceptual components in young children. In contrast, adult results support the hypothesis that motor and perceptual rhythmic preferences reflect a reduced range of common endogenous oscillation, regardless of the task and modality employed in the PPT measure. One hypothesis is that the range of natural, comfortable rates narrows with age, allowing strong relationships between motor and perceptual components to emerge in adulthood, although this result is more evident among males. This narrowing of the tempi range with age implies a significant decline in intra-individual variability. In this regard, rhythmic preferences may be too broad and flexible for younger children to detect the presence of a relationship. In fact, they are less consistent between tasks and even within the same task.

This narrowing of the tempi range and the decline in intra-individual variability with age can also be explained by the acquisition of cognitive resources during childhood. Certain tasks dedicated to PPT measurement may involve greater cognitive demands, resulting in more variability in responses among young children. The cognitive demands intrinsic to PPT measurements seem to be an important avenue of investigation, as inter-group comparisons and appearance of relationships with rhythmic motor preferences change according to task and modality. Hence, the relationships between motor and perceptual rhythmic preferences appear later when the visual modality is employed in PPT measurements. It is well known that children’s temporal processing is less efficient than adults’ in the visual modality, and that the latter is more cognitively demanding^18,19.21^. It is thus possible that these variations in cognitive demands undermine the validity of the measure and delay the emergence of the link between motor and perceptual rhythmic preferences. Further studies are needed to determine the contribution of cognitive development to the onset of the preferred period with age.

Although we mention the emergence of a preferred period with age, our results do not support a strict definition according to which a common endogenous oscillation is reflected by motor and perceptual components, both indicating a marked slowdown with age. First, despite the appearance of relationships between motor and perceptual rhythmic preferences in adulthood, the correspondence is not 1:1. More precisely, there is a mismatch between motor and perceptual rhythmic preferences, as motor rhythmic preferences are generally faster than perceptual ones. This finding also supports a more nuanced definition of the preferred period, as there is no single oscillator common to all rhythmic preferences^16^. Secondly, the present study does not support the hypothesis that a marked slowdown in the preferred period occurs around 8–9 years of age^11^. Our results show that this age group is generally faster and more variable than young adults, in contrast to 11–12-year-olds. This study suggests that a change in rhythmic preferences seems to take place in children aged around 11–12: the relationships between motor and perceptual rhythmic preferences become stronger and significant, while their intra-individual variability does not differ from that of young adults.

The study presents some limitations. Firstly, the number of participants per group and the unequal distribution of male and female participants act as a limitation. In this regard, our sample might not be fully representative of male and female performances within age groups. Additionally, the single trial performed for each standard isochronous sequence in the PPT measurements is also a limitation. Thirdly, the cross-sectional design prevents us from capturing the effect of maturation on the slowing down of rhythmic preferences for an individual. Therefore, it is not possible to determine the extent to which maturation explained the differences between age groups.

In conclusion, our study provides partial confirmation of the preferred period hypothesis. Firstly, the links between motor and perceptual components are evident only in adults. Secondly, a deceleration of the preferred event rate between childhood and adulthood is observed, but this slowing down is notable only in males and is contingent on the task and modality employed in the PPT measurement. Our findings suggests that the range of preferred rhythms narrows with age, becoming less variable in young adulthood, at the expense of a single and consistent, default rhythm.

## Electronic supplementary material

Below is the link to the electronic supplementary material.

Supplementary Material 1

## Data Availability

Datasets collected and analysed during the study are available online: https://osf.io/8hnrp/?view_only=bfcde09b255f4187a882aa7013b4b87a.

## References

[1] Jones, M. R. & Boltz, M. Dynamic attending and responses to time. *Psychol. Rev.* **96**(3), 459–491 (1989). 2756068 10.1037/0033-295x.96.3.459

[2] Jones, M. R. & McAuley, J. D. Time judgments in global temporal contexts. *Percept. Psychophys.* **67**(3), 398–417 (2005). 16119390 10.3758/bf03193320

[3] Drake, C., Jones, M. R. & Baruch, C. The development of rhythmic attending in auditory sequences: attunement, referent period, focal attending. *Cognition* **77**(3), 251–288 (2000). 11018511 10.1016/s0010-0277(00)00106-2

[4] Large, E. W. & Jones, M. R. The dynamics of attending: how people track time-varying events. *Psychol. Rev.* **106**(1), 119–159 (1999).

[5] Boltz, M. G. Changes in internal tempo and effects on the learning and remembering of event durations. *J. Experimental Psychology: Learn. Memory Cognition*. **20**(5), 1154–1171 (1994). 10.1037//0278-7393.18.5.938 1402718

[6] Baudouin, A., Vanneste, S. & Isingrini, M. Age-related cognitive slowing: the role of spontaneous tempo and processing speed. *Exp. Aging Res.* **30**(3), 225–239 (2004). 15487303 10.1080/03610730490447831

[7] Bobin-Bègue, A. & Provasi, J. Rhythmic regulation before 4 years: Effect of an auditory tempo on motor tempo. *L’Année Psychologique*. **108**(4), 631–658 (2008).

[8] Rocha, S., Southgate, V. & Mareschal, D. Infant spontaneous motor tempo. *Dev. Sci.* **24**(2), e13032 (2021). 32860482 10.1111/desc.13032

[9] Turgeon, M., Wing, A. M. & Taylor, L. W. Timing and aging: slowing of fastest regular tapping rate with preserved timing error detection and correction. *Psychol. Aging*. **26**(1), 150–161 (2011). 20973598 10.1037/a0020606

[10] Vanneste, S., Pouthas, V. & Wearden, J. H. Temporal control of rhythmic performance: a comparison between young and old adults. *Exp. Aging Res.* **27**(1), 83–102 (2001). 11205531 10.1080/03610730125798

[11] McAuley, J. D., Jones, M. R., Holub, S., Johnston, H. M. & Miller, N. S. The time of our lives: Life span development of timing and event tracking. *J. Exp. Psychol. Gen.* **135**(3), 348–367 (2006). 10.1037/0096-3445.135.3.348 16846269

[12] Michaelis, K., Wiener, M. & Thompson, J. C. Passive listening to preferred motor tempo modulates corticospinal excitability. *Front. Hum. Neurosci.* **8**, 252 (2014). 24795607 10.3389/fnhum.2014.00252 PMC4006054

[13] Chen, J. L., Penhune, V. B. & Zatorre, R. J. Listening to musical rhythms recruits motor regions of the brain. *Cereb. Cortex*. **18**(12), 2844–2854 (2008). 18388350 10.1093/cercor/bhn042

[14] Bauer, A. K. R., Kreutz, G. & Herrmann, C. S. Individual musical tempo preference correlates with EEG beta rhythm. *Psychophysiology* **52**(4), 600–604 (2015). 25353087 10.1111/psyp.12375

[15] Kliger Amrani, A. & Zion Golumbic, E. Testing the stability of ‘Default’ motor and auditory-perceptual rhythms-A replication failure dataset. *Data Brief.* **32**, 106044 (2020a). 32775563 10.1016/j.dib.2020.106044 PMC7397692

[16] Kliger Amrani, A. & Zion Golumbic, E. Spontaneous and stimulus-driven rhythmic behaviors in ADHD adults and controls. *Neuropsychologia* **146**, 107544 (2020b). 32598965 10.1016/j.neuropsychologia.2020.107544

[17] Trainor, L. J. Are there critical periods for musical development? *Dev. Psychobiol.* **46**(3), 262–278 (2005). 15772967 10.1002/dev.20059

[18] Droit-Volet, S. & Hallez, Q. Differences in modal distortion in time perception due to working memory capacity: a response with a developmental study in children and adults. *Psychol. Res.* **83**(7), 1496–1505 (2019). 29663130 10.1007/s00426-018-1016-5

[19] Droit-Volet, S., Meck, W. H. & Penney, T. B. Sensory modality and time perception in children and adults. *Behav. Process.* **74**(2), 244–250 (2007). 10.1016/j.beproc.2006.09.012 17084041

[20] Lustig, C. & Meck, W. H. Modality differences in timing and temporal memory throughout the lifespan. *Brain Cogn.* **77**(2), 298–303 (2011). 21843912 10.1016/j.bandc.2011.07.007

[21] Zélanti, P. S. & Droit-Volet, S. Auditory and visual differences in time perception? An investigation from a developmental perspective with neuropsychological tests. *J. Exp. Child. Psychol.* **112**(3), 296–311 (2012). 22621934 10.1016/j.jecp.2012.01.003

[22] Hammerschmidt, D. & Wöllner, C. Spontaneous motor tempo over the course of a week: the role of the time of the day, chronotype, and arousal. *Psychol. Res.* **87**(1), 327–338 (2023). 35128606 10.1007/s00426-022-01646-2 PMC8818276

[23] Pollatou, E., Karadimou, K. & Gerodimos, V. Gender differences in musical aptitude, rhythmic ability and motor performance in preschool children. *Early Child. Dev. Care*. **175**(4), 361–369 (2005).

[24] Faul, F., Erdfelder, E., Lang, A. G. & Buchner, A. G*Power 3: a flexible statistical power analysis program for the social, behavioral, and biomedical sciences. *Behav. Res. Methods*. **39**(2), 175–191 (2007). 17695343 10.3758/bf03193146

[25] Cohen, J. *Statistical Power Analysis for the Behavioural Sciences* 2nd edn (Lawrence Erlbaum Associates, 1988).

[26] Psychology Software. Tools, Inc. [E-Prime 3.0]. (2016). Retrieved from https://support.pstnet.com/.

[27] Delacre, M. & Klein, O. Package e ‘mclust’: robust outlier detection. R package version 0.0. *J. Exp. Soc. Psychol.* **49**, 764–766 (2019).

[28] IBM Corp. *IBM SPSS Statistics for Windows, Version 29.0. Armonk* (IBM Corp, Released 2022).

[29] R Core Team. R: A language and environment for statistical computing. https://www.r-project.org/ (R Foundation for Statistical Computing, 2021).

[30] Baayen, R. H., Davidson, D. J. & Bates, D. M. Mixed-effects modeling with crossed random effects for subjects and items. *J. Mem. Lang.* **59**(4), 390–412 (2008).

[31] Krueger, C. & Tian, L. A comparison of the General Linear mixed Model and repeated measures ANOVA using a dataset with multiple Missing Data points. *Biol. Res. Nurs.* **6**(2), 151–157 (2004). 15388912 10.1177/1099800404267682

[32] Tabachnick, B. G. & Fidell, L. S. Using multivariate statistics (7th ed.). (Pearson, 2019).

