# Supplementary material for: A cross sectional investigation of the development of rhythmic preferences with motor and perceptual tests
Source: Sci Rep. 2025 Jan 28;15:3479. doi: 10.1038/s41598-025-87631-2 (PMC11775276; doi:10.1038/s41598-025-87631-2)
Supplement: Supplementary file 1 — Supplementary Material 1 [file 41598_2025_87631_MOESM1_ESM.docx]

**Supplementary Information for**

**A Cross Sectional Investigation of the Development of Rhythmic Preferences with Motor and Perceptual Tests**

Pier-Alexandre Rioux^1,*^ and Simon Grondin^1^

^1^École de Psychology, Université Laval, Québec, Canada
*pier-alexandre.rioux.2@ulaval.ca


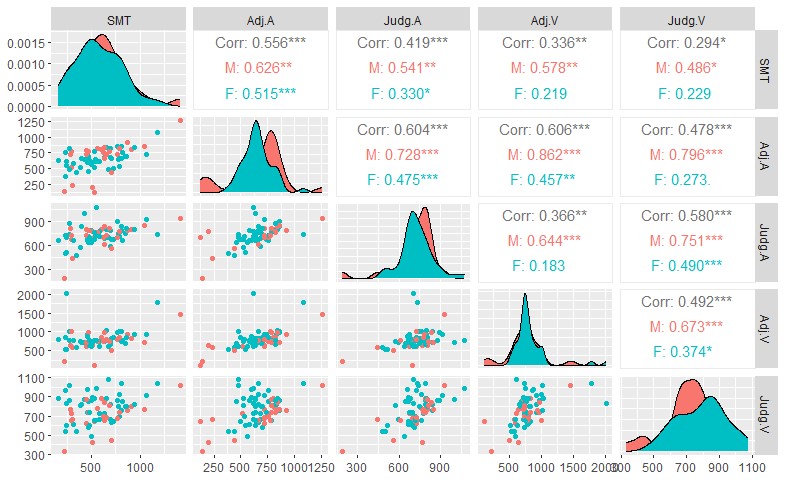


**Figure S1.** Density curves, scatter plots, and correlation matrix of mean ITI (SMT task), adjusted ISI (adjustment task), and intercept (judgment task) for male and female across all groups. Corr. indicates the correlation coefficient for both male and female across all groups. M, Male; F, Female; SMT, Spontaneous Motor Tempo; Adj.A, Adjustment Auditory; Adj.V, Adjustment Visual; Judg.A, Judgment Auditory; Judg.V, Judgment Visual. **p* < .05. ***p* < .01. ****p* < .001.
